# The neurobiology of emotion–cognition interactions: *fundamental questions and strategies for future research*

**DOI:** 10.3389/fnhum.2015.00058

**Published:** 2015-02-17

**Authors:** Hadas Okon-Singer, Talma Hendler, Luiz Pessoa, Alexander J. Shackman

**Affiliations:** ^1^Department of Psychology, University of Haifa, HaifaIsrael; ^2^Functional Brain Center, Wohl Institute of Advanced Imaging, and School of Psychological Sciences, Faculty of Medicine and Sagol School of Neuroscience, Tel Aviv University, Tel AvivIsrael; ^3^Department of Psychology, Neuroscience and Cognitive Science Program, and Maryland Neuroimaging Center, University of Maryland, College Park, College Park, MDUSA

**Keywords:** ACC, amygdala, anxiety, depression, emotion control and regulation, EEG/ERP, fMRI, PFC

## Abstract

Recent years have witnessed the emergence of powerful new tools for assaying the brain and a remarkable acceleration of research focused on the interplay of emotion and cognition. This work has begun to yield new insights into fundamental questions about the nature of the mind and important clues about the origins of mental illness. In particular, this research demonstrates that stress, anxiety, and other kinds of emotion can profoundly influence key elements of cognition, including selective attention, working memory, and cognitive control. Often, this influence persists beyond the duration of transient emotional challenges, partially reflecting the slower molecular dynamics of catecholamine and hormonal neurochemistry. In turn, circuits involved in attention, executive control, and working memory contribute to the regulation of emotion. The distinction between the ‘emotional’ and the ‘cognitive’ brain is fuzzy and context-dependent. Indeed, there is compelling evidence that brain territories and psychological processes commonly associated with cognition, such as the dorsolateral prefrontal cortex and working memory, play a central role in emotion. Furthermore, putatively emotional and cognitive regions influence one another via a complex web of connections in ways that jointly contribute to adaptive and maladaptive behavior. This work demonstrates that emotion and cognition are deeply interwoven in the fabric of the brain, suggesting that widely held beliefs about the key constituents of ‘the emotional brain’ and ‘the cognitive brain’ are fundamentally flawed. We conclude by outlining several strategies for enhancing future research. Developing a deeper understanding of the emotional-cognitive brain is important, not just for understanding the mind but also for elucidating the root causes of its disorders.

Until the 20th century, the study of emotion and cognition was largely a philosophical matter. Although modern perspectives on the mind and its disorders remain heavily influenced by the introspective measures that defined this earlier era of scholarship, the last several decades have witnessed the emergence of powerful new tools for assaying the brain and a remarkable acceleration of research to elucidate the interplay of emotion and cognition (Pessoa, 2013; Braver et al., 2014; Dolcos and Denkova, 2014). The immediate goal of our Special Research Topic was to survey recent advances in understanding how emotional and cognitive processes interact, how they are integrated in the brain, and the implications for understanding the mind and its disorders (Okon-Singer et al., 2014b; **Figure 1**). Here, we consider ways in which this rapidly growing body of work begins to address some more fundamental questions about the nature of cognition–emotion interactions, highlighting key points of consensus. By focusing attention on the most important outstanding questions, we hope to move the field forward. First, we hope that answers provided by our contributors will stimulate discussion. Second, we hope that juxtaposing clear theoretical goals against the current state of the science will motivate new and impactful research. Clearly, our understanding of emotion–cognition interactions remains far from complete. Indeed, we are reminded of Ekman and Davidson’s comment: “There are many promising findings, many more leads, [and] a variety of theoretical stances” (Ekman and Davidson, 1994, p. 3). We conclude by outlining several strategies for enhancing future research. With continuing effort, some of the fundamental questions will be decisively addressed. In some cases, the questions themselves will evolve, as in other areas of the biological sciences.

**FIGURE 1:**
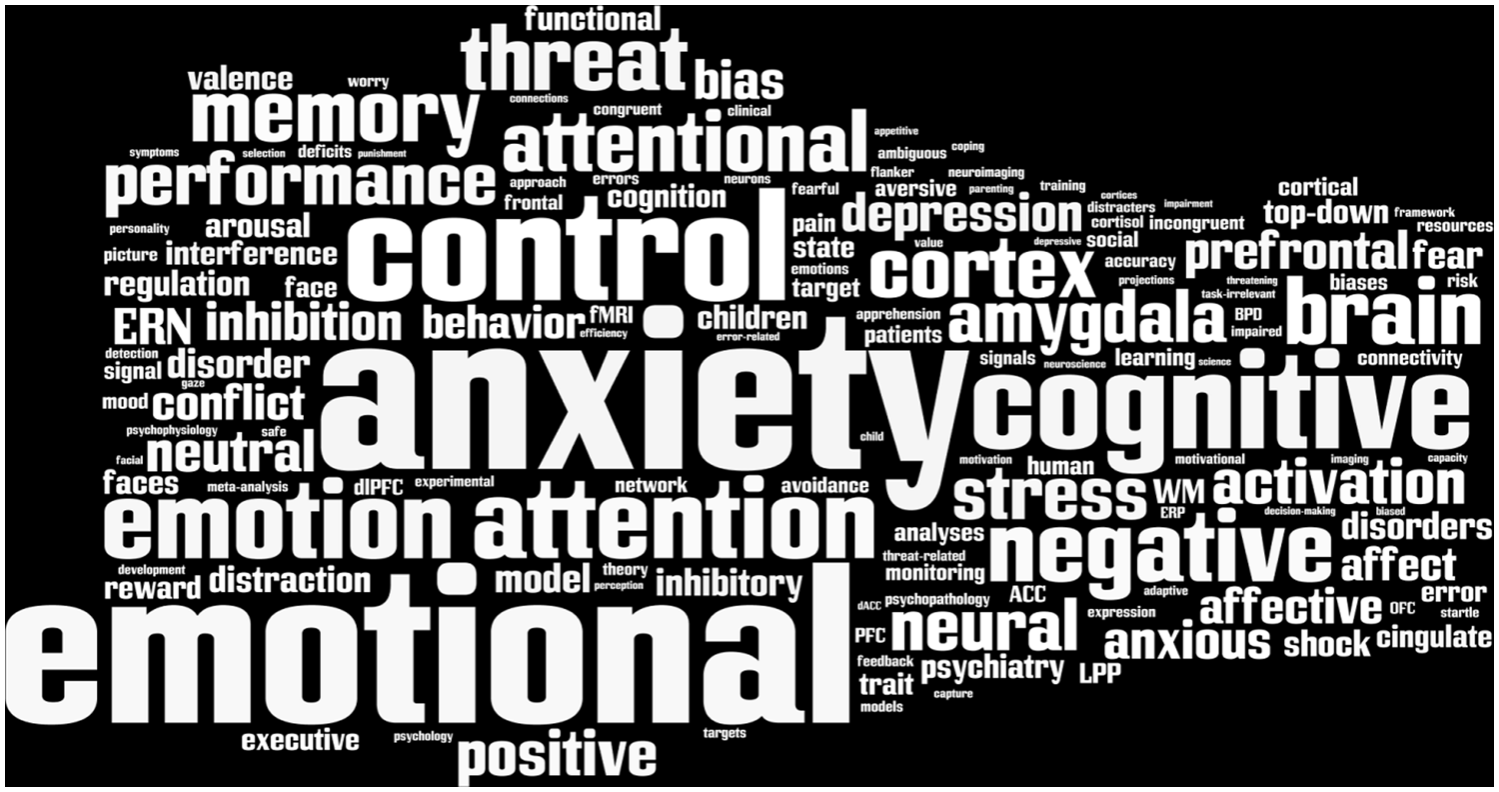
**The top 200 scientific terms used in the Special Research Topic.** The typeface is scaled proportional to the frequency of each term. The figure was generated using http://www.wordle.net.

## HOW DOES EMOTION INFLUENCE COGNITION?

Many of our contributors highlighted evidence that the perception of emotionally-salient stimuli and the experience of emotional states can profoundly alter cognition.

### EMOTIONAL CUES GRAB EXOGENOUS ATTENTION AND MODULATE ENDOGENOUS ATTENTION

There is abundant evidence that emotionally-salient cues—snakes, spiders, and angry faces—strongly influence attention (e.g., Siman-Tov et al., 2009; Lerner et al., 2012; Pourtois et al., 2013; Carretié, 2014) the ability to selectively respond to relevant aspects of the environment while inhibiting potential sources of distraction and competing courses of action (Desimone and Duncan, 1995; Miller and Cohen, 2001). The focus of attention is determined by the pervasive competition between exogenous (often termed ‘stimulus-driven’ or ‘bottom–up’) and endogenous (often termed ‘goal-directed’ or ‘top–down’ attention) mechanisms (Egeth and Yantis, 1997).

With respect to exogenous attention, a number of contributors describe new evidence that emotionally-charged cues are more attention-grabbing than neutral cues and highlight recent efforts to specify the mechanisms underlying this bias (Holtmann et al., 2013; McHugo et al., 2013; Peers et al., 2013; Stollstorff et al., 2013). Along the way, McHugo et al. (2013) provide a useful tutorial on methods for quantifying the capture of attention by emotional cues (e.g., dot-probe, emotional attentional blink).

Importantly, attention can also be guided in an endogenous fashion by internal goals (e.g., rules, instructions, and plans) as well as moods and motivational states (e.g., feeling anxious or hungry). Mohanty and Sussman (2013) discuss evidence demonstrating that emotion and motivation can guide attention to congruent cues (e.g., food when hungry). In particular, they show that subcortical regions proximally involved in determining value and orchestrating emotional states (e.g., amygdala, substantia nigra) can facilitate endogenous attentional processes implemented in frontoparietal regions and can strengthen activation in relevant sensory regions (e.g., face-selective regions of the fusiform gyrus when anticipating an angry face). This extended network, encompassing sensory, attentional, and emotional circuits, facilitates the rapid detection of emotionally-significant information.

### ATTENTIONAL BIASES TO EMOTIONAL CUES ARE PLASTIC

Anxious individuals tend to allocate excess attention to threat and there is evidence that this cognitive bias causally contributes to the development and maintenance of anxiety disorders (Bar-Haim et al., 2007; Hakamata et al., 2010; MacLeod and Mathews, 2012; Singer et al., 2012; Van Bockstaele et al., 2013; MacLeod and Clarke, 2015). Extreme anxiety and behavioral inhibition often emerges early in development (Fox et al., 2005a; Blackford and Pine, 2012; Fox and Kalin, 2014), raising important questions about the degree to which childhood attentional biases to threat are plastic and can be influenced by early experience (Shechner et al., 2012; Bar-Haim and Pine, 2013; Henderson et al., 2014; MacLeod and Clarke, 2015).

Here, Kessel et al. (2013) provide tantalizing correlative evidence that emotional biases in attention are influenced by caregiver style. Using an innovative combination of behavioral and electrophysiological techniques, they show that although temperamentally inhibited children allocate more attention to aversive cues, this is reduced among the offspring of parents who rely on encouragement, affection, and appreciation to reinforce positive behavior. A key challenge for future research will be to test whether targeted interventions aimed at cultivating more salubrious parenting styles have similar consequences. Prospective designs (e.g., before and after exposure to a negative life event or trauma) would provide another powerful approach for understanding the plasticity of emotional attention (Admon et al., 2009, 2012).

### EMOTION EXERTS PERSISTENT EFFECTS ON ATTENTION

Emotions are often conceptualized as fleeting and most imaging and psychophysiological studies of emotion focus on transient responses to punctate emotional challenges. Yet, there is growing evidence that emotions can have lingering consequences for cognition and behavior (Davidson, 2004; Suls and Martin, 2005; Hajcak and Olvet, 2008; Qin et al., 2009).

Here, for example, Vaisvaser et al. (2013) combined serial measures of emotional state, neuroendocrine activity, and resting-state brain activity to demonstrate that alterations in amygdala–hippocampal functional connectivity persist for more than 2 h following exposure to intense social stress. Along conceptually similar lines, Morriss et al. (2013) use electrophysiological techniques to show that endogenous attention is potentiated for several seconds following brief emotional challenges (i.e., standardized emotional images).

Several threads of evidence highlight the importance of understanding the mechanisms that govern variation in the speed of recovery from emotional perturbation. In particular, individual differences in emotional recovery (a) strongly predict personality traits, such as neuroticism, that confer increased risk of developing psychopathology (e.g., Blackford et al., 2009; Schuyler et al., 2014); and (b) are sensitive to adversity and chronic stress exposure, two other well-established risk factors (Lapate et al., 2014). An important challenge for future research will be to identify the neural circuitry and molecular pathways that support the enduring effects of emotion on endogenous attention and to clarify the intermediate processes that link variation in emotional recovery to mental health and disease.

### DISTRACTING EMOTIONAL CUES READILY PENETRATE THE GATE PROTECTING WORKING MEMORY

Endogenous attention is tightly linked with working memory (Postle, 2006; D’Esposito and Postle, 2014; Sreenivasan et al., 2014). The transient representation of task-sets, goals, and other kinds of information in working memory plays a crucial role in sustaining goal-directed attention and guiding behavior in the face of potential distraction (Miller and Cohen, 2001). In short, information held in working memory is a key determinant of our momentary thoughts, feelings, and behavior. Importantly, the capacity of working memory is strongly determined by the ability to filter or gate irrelevant information (Vogel et al., 2005; McNab and Klingberg, 2007; Awh and Vogel, 2008).

Here, Stout et al. (2013) used a well-established electrophysiological marker of working memory storage (i.e., contralateral delay activity; Vogel and Machizawa, 2004) to show that threat-related distractors (i.e., task-irrelevant fearful faces) are stored in working memory and that this filtering inefficiency is exaggerated in dispositionally-anxious individuals. Once in working memory, emotional information is poised to hijack endogenous attention and other kinds of top–down control mechanisms. From a psychiatric perspective, this emotional gating deficit may help to explain the persistence of heightened negative affect (e.g., anxiety, sadness) among patients with emotional disorders (Grupe and Nitschke, 2013; Cohen et al., 2014; Stout et al., 2014). An important challenge for future studies will be to use hemodynamic imaging techniques, such as fMRI, to clarify the neural circuitry underlying emotional gating deficits. A variety of evidence suggests that the pulvinar may play an important role (Pessoa and Adolphs, 2010; Arend et al., 2014).

### DISTRACTING EMOTIONAL CUES DISRUPT COGNITIVE CONTROL AND WORKING MEMORY

Classically, cognition and emotion have been viewed as oppositional forces (Damasio, 2005a; Okon-Singer et al., 2007, 2011; Shackman et al., in press). From this perspective, moods and other kinds of emotional states are responsible for short-circuiting cognition.

Consistent with this view, Kalanthroff et al. (2013) show that emotional distractors disrupt cognitive control. Cognitive control encompasses the range of processes (e.g., endogenous attention, inhibition, and learning) that are engaged when habitual responses are not sufficient to sustain goal-directed behavior, as in stop-signal, go/no-go, Stroop, and Eriksen flanker tasks (Shackman et al., 2011b). Here, the authors demonstrate that the brief presentation of emotional images disrupts performance in the stop-signal task, a widely used index of inhibitory control (see also Pessoa et al., 2012).

Likewise, Iordan et al. (2013) review evidence that emotional distractors disrupt working memory. Converging with other work focused on emotion-related distraction (Bishop, 2007; Etkin, 2012; Bishop and Forster, 2013; Etkin et al., 2013; Okon-Singer et al., 2014a; van Ast et al., 2014), they suggest that degraded performance reflects two processes: (a) increased engagement of regions involved in processing socio-emotional information and orchestrating emotional expressions (e.g., amygdala), and (b) a reduction of delay-spanning activity in frontoparietal cortex.

### EMOTION STRENGTHENS SOME COGNITIVE PROCESSES WHILE WEAKENING OTHERS

With the ascent of evolutionary theory in the 19th century (Darwin, 1872/2009, 1872), many scientists adopted the view that emotions are functional and enhance fitness (Susskind et al., 2008; Todd et al., 2012; Sandi, 2013; Schwabe and Wolf, 2013; Todd and Anderson, 2013); in short, that emotions are more adaptive than not and “that there is typically more cooperation than strife” between emotion and cognition (Levenson, 1994).

Consistent with this more nuanced perspective, the contributions from Clarke and Johnstone (2013), Morriss et al. (2013), Robinson et al. (2013a, 2013b), Vytal et al. (2013) provide evidence that experimentally-elicited anxiety facilitates some kinds of information processing, while degrading others. In particular, they provide considerable evidence that anxiety: (a) enhances vigilance, potentiating early sensory cortical responses to innocuous environmental stimuli, increasing the likelihood that emotionally salient information will be detected; and (b) disrupts working memory.

The molecular basis of emotion’s deleterious impact on working memory is reviewed by Shansky and Lipps (2013). Building on pioneering work by Arnsten and Goldman-Rakic (1998) and Arnsten (2009), the authors describe evidence that stress strongly influences catecholamine (i.e., dopamine and norepinephrine) and glucocorticoid levels in the prefrontal cortex (PFC) in ways that degrade delay-spanning neuronal activity.

Shansky and Lipps (2013) also describe important new evidence that sex hormones, such as estrogen, can exacerbate the impact of stress on prefrontal function. Along these lines, Sacher et al. (2013) review human imaging studies showing that the structure and function of brain circuits involved in emotion generation and regulation are strongly and dynamically modulated by cyclic fluctuations in sex hormones (see also Sacher et al., 2012). Taken together, these observations underscore the plasticity of emotion–cognition interactions and provide promising clues about the origins of well-established sex differences in the prevalence of stress-related disorders, such as anxiety and depression (Kessler et al., 2012; Kendler and Gardner, 2014).

### EMOTIONAL STATES PROMOTE MOOD-CONGRUENT THOUGHTS AND ACTIONS

Moods and other, more transient emotional states tend to encourage congruent thoughts and actions (e.g., Lerner et al., 2015), a process that is necessarily mediated by enduring changes in brain activity and connectivity (cf. Vaisvaser et al., 2013). Here, Van Dessel and Vogt (2012) demonstrate that mood increases the amount of attention allocated to mood-congruent cues. Schick et al. (2013) provide evidence that individuals at risk for developing depression interpret motivationally ambiguous cues in a less positive light. Harlé et al. (2013) describe a novel Bayesian computational framework for understanding the mechanisms underlying mood-congruency effects. An important advantage of this framework is that it generates explicit mechanistic hypotheses. For example, the model predicts that anxiety facilitates behavioral avoidance because it leads to inflated expectations about the need for avoidant behavior and increased expectations of punishment or error. Furthermore, fitting model parameters to observable behavior affords an opportunity to identify the underlying determinants of mood-congruency effects in healthy and clinical populations.

### EMOTIONAL TRAITS INFLUENCE COGNITIVE PERFORMANCE, EVEN WHEN EMOTIONAL CUES, AND CHALLENGES ARE ABSENT

Emotional traits are often conceptualized as diatheses for emotional states (Matthews et al., 2009). Thus, individuals with high levels of neuroticism or negative emotionality are thought to be prone to exaggerated anxiety in the face of trait-relevant cues, contexts, and challenges (e.g., punishment, negative feedback), as illustrated in the contributions from Kessel et al. (2013), Moser et al. (2013), and Proudfit et al. (2013). Yet, a considerable body of neurophysiological evidence indicates that emotional traits are embodied in the on-going activity and connectivity of the brain (Canli et al., 2005; Fox et al., 2008; Shackman et al., 2009; Rohr et al., 2013; Birn et al., 2014a,b). Likewise, the sustained levels of heightened vigilance and distress characteristic of individuals with anxiety disorders are most apparent in the absence of clear and imminent threat (Davis et al., 2010; Lissek, 2012; Grupe and Nitschke, 2013). These observations raise the possibility that emotional traits could influence cognition in the absence of explicit emotional distraction or challenge (Watson and Clark, 1984; Bolger and Schilling, 1991; Suls and Martin, 2005).

Here, Berggren et al. (2013) provide compelling evidence that trait anxiety is associated with degraded cognitive control, indexed using an anti-saccade task under load. This new observation adds to a growing literature showing that “hot” emotional traits can influence “cold” cognition (Shackman et al., 2006; Eysenck et al., 2007; Bishop, 2009; Berggren and Derakshan, 2013, 2014; Cavanagh and Shackman, 2014), a point that we develop more fully in the subsequent section focused on the integration of emotion and cognition.

## HOW DOES EMOTION INFLUENCE EMOTION?

An important but rarely addressed question in psychology and psychiatry concerns the potential influence of emotions on one another and concomitant motivational states. For example, are we less likely to experience excitement or joy on a day where we’re feeling frazzled, depressed, or worn out (Arnsten, 1998, 2009; Pizzagalli, 2014)?

### EMOTION ALTERS REINFORCER SENSITIVITY

Building on earlier work by Bogdan and Pizzagalli (2006), Pizzagalli et al. (2007), Bogdan et al. (2010), and Berghorst et al. (2013) demonstrate that experimentally-elicited anxiety selectively reduces sensitivity to reward, suggesting a mechanism that may contribute to the high rate of comorbidity between anxiety and anhedonia (Southwick et al., 2005). Notably, this effect was only observed in the subset of subjects who were most responsive to the anxiety induction (i.e., threat of noxious electric shock). Given evidence that many individuals will never experience a mood or anxiety disorder (Kessler et al., 2012), this paradigm may provide a means of identifying those at greatest risk. Methodologically, this observation underscores the necessity of including independent measures of emotion in studies of emotion–cognition interactions (Shackman et al., 2006).

## HOW DOES COGNITION INFLUENCE AND REGULATE EMOTION?

Humans frequently regulate their emotions and they do so using a variety of implicit and explicit cognitive strategies (Gross, 1998a,b; Gross and Thompson, 2007; Gross et al., 2011; Webb et al., 2012; Okon-Singer et al., 2013). Implicit strategies are unintentional and appear to occur without effort or insight. In contrast, explicit strategies are voluntary and demand a degree of effortful control.

Several contributors to our Special Research Topic described new insights into the mechanisms supporting the cognitive regulation of emotion and the role of emotion regulation in psychiatric disorders, such as depression.

### ATTENTION REGULATES EMOTION

Perhaps the most basic strategy for reducing distress is attentional avoidance; that is, to simply look away from the source of distress (Xing and Isaacowitz, 2006). Overt attentional redeployment is a potent means of regulating the engagement of subcortical structures, such as the amygdala, that play a key role in orchestrating emotional states (Pessoa et al., 2002; Dalton et al., 2005; Dalton et al., 2007; van Reekum et al., 2007; Urry, 2010; Okon-Singer et al., 2014a).

Here, Aue et al. (2013b) employed an innovative combination of eyetracking, psychophysiology, and fMRI to explore visual avoidance in spider phobics. Taking an individual differences approach, they demonstrate that enhanced activation in the amygdala and dorsal striatum to spider images was predictive of increased visual avoidance among arachnophobes. Peripheral measures of autonomic arousal showed a similar pattern, suggesting that arachnophobes endogenously redirect attention as a means of regulating their extreme fear, a strategy that might be non-adaptive in the long term (Grupe and Nitschke, 2013). A key challenge for future research will be to clarify the order of these effects (i.e., fear → attention avoidance → reduced fear), perhaps by leveraging the millisecond temporal resolution afforded by facial electromyography (e.g., Lee et al., 2009; Heller et al., 2014). Elucidating the mechanisms supporting the recursive interplay of emotion and attention and the mutual influences of different processing biases (Aue et al., 2013a) would inform our understanding of disorders, like post-traumatic stress, that are characterized by dysregulated emotion and aberrant attention to emotionally-salient cues (e.g., Admon et al., 2013; Wald et al., 2013) and set the stage for developing improved interventions (MacLeod and Mathews, 2012; Bar-Haim and Pine, 2013; MacLeod and Clarke, 2015).

### THE CHOICE OF COGNITIVE REGULATION STRATEGY DEPENDS ON THE SITUATION

Sheppes and Levin (2013) emphasize that humans frequently use effortful cognitive strategies to cope with and regulate their emotions (e.g., Egloff et al., 2006; Ehring et al., 2010). For example, they may try to distract themselves or they may try to reappraise the situation in a more positive light. Sheppes and Levin (2013) provide evidence that not only do individuals have the capacity to flexibly choose emotion regulation strategies, but that they do so in ways that are strongly influenced by the emotional context (e.g., choosing to reappraise when presented with mild negative pictures, and to distract themselves in face of highly aversive stimulation).

### WORKING MEMORY REGULATES EMOTION

Some strategies for regulating emotional distress, such as reappraisal, require the effortful maintenance of an explicit regulatory goal. Rolls (2013) reviews evidence suggesting that this critically depends on working memory. More broadly, he suggests that goals, attentional sets, and other kinds of declarative knowledge held in working memory play a central role in regulating the output of emotional systems.

## HOW ARE EMOTION AND COGNITION INTEGRATED?

Humans tend to experience cognition and emotion as fundamentally different. Emotion is infused with feelings of pleasure or pain and manifests in readily discerned changes in the body, whereas cognition often appears devoid of substantial hedonic, motivational, or somatic features. These apparent differences in phenomenological experience and peripheral physiology led many classical scholars to treat emotion and cognition as distinct mental faculties (de Sousa, 2014; Schmitter, 2014). But contemporary theorists have increasingly rejected the claim that emotion and cognition are categorically different (Damasio, 2005b; Duncan and Barrett, 2007; Lindquist and Barrett, 2012; Barrett and Satpute, 2013; Pessoa, 2013), motivated in part by recent imaging evidence demonstrating the overlap of emotional and cognitive processes in the brain (e.g., Shackman et al., 2011b; Raz et al., 2012, 2014). The neural integration of emotion and cognition should not be surprising—after all, the human brain did not evolve to optimize performance on laboratory measures of ‘cold’ cognition or to passively respond to experimental manipulations of emotion, such as threat of shock. Our brain, like that of other animals, is the product of evolutionary pressures that demanded neural systems capable of using information about pleasure and pain, derived from stimuli saturated with hedonic and motivational significance, to adaptively regulate attention, learning, somatic arousal, and action.

A number of contributors highlighted advances in our understanding of the neural mechanisms that serve to integrate emotion and cognition.

### CANONICAL TERRITORIES OF THE ‘COGNITIVE’ BRAIN REGULATE EMOTION

The dorsolateral prefrontal cortex (dlPFC) is a canonically ‘cognitive’ region of the brain, well known for its critical role in reasoning and higher cognition (e.g., endogenous attention, working memory, and cognitive control; Roberts et al., 1998; Miller and Cohen, 2001; D’Esposito and Postle, 2014). Yet, there is growing evidence that the dlPFC plays a key role in the top–down control of emotion and motivated behavior (Fox et al., 2005b; Koenigs et al., 2008; Zaretsky et al., 2010; Buhle et al., 2013; Frank et al., 2014; Treadway et al., 2014).

Here, Clarke and Johnstone (2013) and Iordan et al. (2013) provide tantalizing, albeit correlational, evidence that dlPFC acts to protect the contents of working memory from emotional distraction. This converges with work by Peers et al. (2013) and Stollstorff et al. (2013) indicating that dlPFC plays a key role in regulating the focus of attention in the face of potentially distracting emotional cues.

Rolls (2013) extends this perspective to decision-making, arguing that behavior reflects a pervasive, dynamic competition between two kinds of brain systems: (a) emotional systems, including circuits centered on the amygdala and ventral striatum, that have been genetically programmed by our phylogenetic history (e.g., fear elicited by danger, joy elicited by sweets and fat); and (b) cognitive systems, such as the frontoparietal network, that are informed by our ontogenetic history and governed by our declarative knowledge and explicit goals (i.e., *pick the healthy orange, not the unhealthy candy bar*; cf. Hare et al., 2008, 2009). Rolls emphasizes that the lateral PFC can override the output of emotion circuitry, biasing behavior in favor of our explicit goals. John et al. (2013) articulate a complementary perspective, reviewing evidence that the PFC and amygdala functionally interact via a complex anatomical network of recurrent cortical and thalamic projections and intra-amygdalar microcircuits (see also Pessoa and Adolphs, 2010; Pessoa, 2012; Pessoa et al., 2012; Birn et al., 2014a,b; Treadway et al., 2014).

Evidence linking the dlPFC to mood and anxiety disorders, as in the papers contributed by Crocker et al. (2013) and Warren et al. (2013), underscores the importance of developing a more sophisticated understanding of the role played by ‘cognitive’ regions in normal and disordered emotion.

### CANONICAL TERRITORIES OF THE ‘COGNITIVE’ BRAIN ARE REGULATED BY EMOTION

Regulation is a two-way street. Just as ‘cognitive’ systems (e.g., dlPFC) regulate emotion, ‘emotion’ systems (e.g., amygdala) are well positioned to regulate ‘cognitive’ systems via their influence over the brainstem neurotransmitter systems that govern the quality of information processing (e.g., neuronal signal-to-noise) in cortical regions, as highlighted in the review contributed by Shansky and Lipps (2013). Via these mechanisms, the amygdala is endowed with the capacity to transiently assume enhanced control over attention and behavior in situations that favor immediate responses over slower, more deliberate reasoning (Davis and Whalen, 2001; Arnsten, 2009).

### ADAPTIVE AND MALADAPTIVE BEHAVIOR REFLECTS THE INTEGRATED CONTRIBUTIONS OF EMOTION AND COGNITIVE CONTROL

Oftentimes, cognitive control is associated with laboratory tasks that require the detection and adjudication of response conflict, as with incongruent trials of the Stroop, Eriksen Flanker, and go/no-go tasks. Yet, it is clear that control processes are engaged by a much broader range of cognitive and emotional challenges (e.g., Pochon et al., 2008; Shenhav et al., 2013). In particular, control is engaged when there is uncertainty about the optimal course of action (e.g., probabilistic learning), when potential actions are associated with the possibility of error or punishment, or when there is competition between alternative courses of action (e.g., flee/freeze, go/no-go). These features are hallmarks of dangerous environments, both in the real world and in laboratory studies of fear, anxiety, and pain. Consequently, it has long been thought that control processes are engaged in threatening environments in order to monitor risk, optimize learning, and avoid potentially catastrophic actions (Norman and Shallice, 1986; Gray and McNaughton, 2000). These theoretical considerations raise the possibility that the neural circuitry underlying ‘cognitive’ control also contributes to the negative emotions elicited by potential threat. Indeed, there is compelling evidence from functional imaging studies that negative affect and cognitive control paradigms consistently activate an overlapping region of the midcingulate cortex (MCC; Shackman et al., 2011b; Lin et al., 2014). This overlap is consistent with anatomical evidence suggesting that the MCC represents a hub where information about pain, threat, and other more abstract forms of potential punishment and negative feedback are synthesized into a biasing signal that modulates regions involved in expressing fear and anxiety, executing goal-directed behaviors, and biasing the focus of selective attention (Shackman et al., 2011b; Cavanagh and Shackman, 2014). Taken together, these observations suggest that anxiety and other emotions are tightly integrated with control processes implemented in the MCC and other brain regions.

Along these lines, Morrison et al. (2013) show that even simple, phylogenetically-ancient kinds of motivated behavior, such as the reflexive withdrawal from pain or the learned avoidance of pain-related contexts, are dynamically shaped by complex, hierarchically-organized networks of feedforward and feedback connections that serve to integrate ‘emotional’ (e.g., value, risk) and ‘cognitive’ computations (e.g., prediction error, attention allocation, action selection) in ways that support adaptive behavior (for convergent perspectives, see the contributions from Rolls, 2013, and John et al., 2013).

Dreisbach and Fischer (2012) describe other evidence consistent with this integrative perspective. In particular, they show that ‘cognitive’ conflict is aversive. This converges with a growing body of evidence demonstrating that conflict and other prompts for increased control (e.g., errors, punishment), are experienced as unpleasant and facilitate avoidance (Botvinick, 2007; Kool et al., 2010; Dreisbach and Fischer, 2012; Schouppe et al., 2012; Lindström et al., 2013; Proudfit et al., 2013; Shenhav and Buckner, 2014).

If negative emotions are indeed integrated with control processes, we would expect that anxiety and control should covary. That is, one would expect a degree of functional convergence between measures of anxiety and control-related activity in the MCC or other regions (i.e., convergent validity; Campbell and Fiske, 1959). Consistent with this possibility, Moser et al. (2013) provide compelling meta-analytic evidence that error-related signals generated in the MCC are enhanced among anxiety patients and individuals with heightened negative emotionality. This indicates that negative emotionality, a fundamental dimension of childhood temperament and adult personality (Caspi et al., 2005), involves systematic differences in the way that the brain responds to prompts for cognitive control.

McDermott et al. (2013) describe important new evidence, gleaned from the study of Romanian orphans, that MCC control signals are plastic. In particular, they demonstrate that MCC-generated control signals are profoundly shaped by early experience in ways that confer risk or resilience for later socio-emotional problems. This underscores the need to clarify the neurodevelopmental mechanisms that serve to integrate emotion and cognition in the laboratory and in daily life.

## UNDERSTANDING THE INTERPLAY OF EMOTION AND COGNITION: STRATEGIES FOR FUTURE RESEARCH

Despite substantial progress, a number of important questions about the interaction of emotion and cognition remain unanswered. In this final section, we highlight three strategies for enhancing research in the cognitive-affective sciences (for more general recommendations about best research practices, see Button et al., 2013a,b,c; David et al., 2013; Chalmers et al., 2014; Ioannidis et al., 2014a,b).

### UNDERSTANDING THE SIGNIFICANCE OF EMOTIONAL-COGNITION INTERACTIONS IN THE LABORATORY REQUIRES MORE SOPHISTICATED MEASURES OF BEHAVIOR IN THE REAL WORLD

Most investigations of emotion, cognition, and their interplay rely on a small number of well-controlled, but highly artificial paradigms for manipulating emotion and cognition (e.g., static aversive images and threat of shock to elicit anxiety; Coan and Allen, 2007). Although these methods have afforded a number of critical insights, their real-world significance remains poorly understood. For example, are attentional biases to threat, as indexed by the dot-probe or other laboratory assays, predictive of elevated behavioral inhibition or distress in daily life? Is amygdala activation to fearful faces predictive of heightened social reticence or risk avoidance outside the scanner (see Admon et al., 2009 for preliminary affirmative evidence)? Does the eliciting stimulus (e.g., faces or aversive images) matter? Are measures of functional connectivity or network-based metrics (e.g., node centrality; cf. McMenamin et al., 2014) more predictive than regional activation of behavior in the real world?

Given the limitations of ambulatory measures of brain activity—there is no ‘fMRI helmet’ as yet—addressing these fundamental questions requires pairing assays of brain and behavior obtained in the laboratory with measures of thoughts, feelings, and behavior obtained in the field. Recent work combining fMRI with ecological momentary assessment (EMA) techniques, in which surveys are repeatedly delivered to participants’ mobile devices, highlights the value of this approach for identifying the neural systems underlying naturalistic variation in mood and behavior, a central goal of psychology, psychiatry, and the behavioral neurosciences (Forbes et al., 2009; Berkman and Falk, 2013; Lopez et al., 2014; Wilson et al., 2014). The widespread dissemination of smart phone technology affords additional, largely unrealized opportunities for objectively and unobtrusively quantifying daily behavior (e.g., assessments of activity and context based on accelerometer and geographical positioning system data (Gosling and Mason, 2015). In short, combining EMA with laboratory assays provides a critical means of testing theoretical validity and clinical relevance (e.g., *does activation of the ventral striatum support craving and approach?*), a novel strategy for assessing and dissociating the functional significance of new assays and derivative measures (e.g., functional connectivity between the striatum and PFC), and an impetus for the development of laboratory probes that more closely resemble the challenges we routinely encounter in life (e.g., appetitive social cues and temptations).

### UNDERSTANDING THE INTERPLAY OF EMOTION AND COGNITION REQUIRES A DYNAMIC NETWORK PERSPECTIVE

Emotion and cognition emerge from the dynamic interactions of large-scale brain networks. Put simply, fear, joy, attention, working memory, and other psychological constructs cannot be mapped to isolated brain regions because no one region is both necessary and sufficient. Likewise, similar profiles of impairment can emerge from damage to different regions located within in the same functional network (Karnath and Smith, 2014; Oler et al., in press). This is not a new or contentious idea; pioneers like Mesulam, Goldman-Rakic, and LeDoux highlighted the importance of distributed neural circuits more than two decades ago and there is widespread agreement amongst basic and translational researchers (Goldman-Rakic, 1988; LeDoux, 1995; Mesulam, 1998; Bullmore and Sporns, 2012; LeDoux, 2012; Uhlhaas and Singer, 2012; Anticevic et al., 2013).

Thus, understanding the interplay of emotion and cognition requires that we accelerate the transition from localization strategies (i.e., mapping isolated brain structures to function; sometimes termed ‘neo-phrenology’) to a network-centered approach. This will require harnessing the kinds of analytic tools (e.g., functional connectivity fingerprinting, graph-theoretic and machine-learning approaches) that are necessary for elucidating how adaptive and maladaptive behavior emerges from functional coalitions of brain regions (Kinnison et al., 2012; Raz et al., 2012, 2014; Anticevic et al., 2013; McMenamin et al., 2014; Uddin et al., 2014). A key challenge for future research will be to harness new techniques (e.g., EEG/fMRI fusions, sliding window analyses of functional connectivity, EEG source models of connectivity) for understanding how network activity dynamically changes across the broad range of time scales on which emotion and cognition interact (Pessoa and Adolphs, 2010; Shackman et al., 2011a; Johnson et al., 2012; Raz et al., 2012, 2014).

Computationally explicit strategies (i.e., where quantitative parameters of an abstract computational model are fit to behavioral or physiological measures), already common in the neuroeconomics literature, and information-based approaches, such as multivoxel pattern analysis (MVPA), that are increasingly common in the cognitive neuroscience literature, provide powerful tools for discovering the functional significance of regions and networks associated with emotional and cognitive perturbations and disorders (e.g., Hartley and Phelps, 2012; Montague et al., 2012; Lewis-Peacock and Norman, 2013). For example, traditional univariate fMRI analyses use regression to predict the activity of voxels, one-by-one, given some mental state (e.g., experiencing pain). While this strategy has proven enormously generative, it does not provide strong evidence as to whether overlapping patterns of fMRI activation (e.g., during physical and social pain; Wager et al., 2013; Woo et al., 2014) reflect the same mental representation. MVPA provides a means of addressing this problem. MVPA classifies mental states given a pattern of activity across voxels; in effect, treating each voxel as a weighted source of information about mental state. This contributes to the identification of the combinatorial code (i.e., pattern of activity across voxels) instantiating a particular mental state (e.g., experiencing anxiety) and to test whether that neural signature is reinstated at other times (e.g., performing a cognitive control task), an essential step in elucidating the functional contributions of territories that are commonly recruited by cognitive and emotional challenges (e.g., dlPFC, MCC, anterior insula).

Embracing a network perspective also reminds us that the functional circuitry underlying the interplay of emotion and cognition is likely to be complex and need not recapitulate the simpler pattern of direct projections revealed by invasive anatomical tracing techniques [cf. the contributions from John et al. (2013), Morrison et al. (2013), and Rolls (2013)]. Indeed, there is ample evidence of robust functional connectivity between brain regions that lack direct structural connections and increasing evidence that regulatory signals can rapidly propagate across complex, indirect pathways in ways that enable emotion (e.g., motivational salience or value) to be integrated with perception and other kinds of on-going information processing (Vincent et al., 2007; Ekstrom et al., 2008; Honey et al., 2009; Pessoa and Adolphs, 2010; Adachi et al., 2012; Birn et al., 2014a). Deciphering the functional significance of this ‘connectomic’ complexity is likely to require more advanced analytic approaches, such as probabilistic machine-learning techniques (Murphy, 2012). The combination of ongoing advances in computational methods as well as developments in brain imaging acquisition techniques (e.g., those supported by the U.S. BRAIN initiative) will undoubtedly contribute to these efforts.

### UNDERSTANDING THE INTERPLAY OF EMOTION AND COGNITION REQUIRES MECHANISTIC RESEARCH

Most of the contributors to the Special Research Topic used non-invasive techniques, such as fMRI, to trace associations between emotion and cognition, on the one hand, and brain function on the other. Aside from unresolved questions about the origins and significance of the measured signals (e.g., Logothetis, 2008), the most important limitation of these techniques is that they do not address causation. A crucial challenge for future studies is to develop a mechanistic understanding of the distributed networks that support the interplay of emotion and cognition. This can be achieved by combining mechanistic techniques (e.g., optogenetics) or invasive analyses of neuromolecular pathways in animal models with the same whole-brain imaging strategies routinely applied in humans (Borsook et al., 2006; Lerman et al., 2007; Fox et al., 2010, 2012; Lee et al., 2010; Desai et al., 2011; Casey et al., 2013; Narayanan et al., 2013; Roseboom et al., 2014). Similar strategies can be used with patients with circumscribed brain damage (e.g., Nomura et al., 2010; Gratton et al., 2012; Motzkin et al., 2014). Combining fMRI or EEG with non-invasive perturbation techniques (e.g., transcranial magnetic stimulation or transcranial direct current stimulation) or pharmacological manipulations provides another opportunity for understanding how regional changes in brain activity alter network function and, ultimately, behavior (Paulus et al., 2005; Guller et al., 2012; Chen et al., 2013; Reinhart and Woodman, 2014). Prospective longitudinal designs represent another fruitful approach to identifying candidate mechanisms, especially in relation to the development of neuropsychiatric disorders (Admon et al., 2013).

## CONCLUSION

The last decade has witnessed an explosion of interest in the interplay of emotion and cognition. The research embodied in this Special Research Topic highlights the tremendous advances that have already been made. In particular, this work demonstrates that emotional cues, emotional states, and emotional traits can strongly influence key elements of on-going information processing, including selective attention, working memory, and cognitive control. Often, this influence persists beyond the duration of transient emotional challenges, perhaps reflecting slower changes in neurochemistry. In turn, circuits involved in attention and working memory contribute to the voluntary regulation of emotion. The distinction between the ‘emotional’ and the ‘cognitive’ brain is blurry and context-dependent. Indeed, there is compelling evidence that territories (e.g., dlPFC, MCC) and processes (e.g., working memory, cognitive control) conventionally associated with cognition play a central role in emotion. Furthermore, putatively emotional and cognitive regions dynamically influence one another via a complex web of recurrent, often indirect anatomical connections in ways that jointly contribute to adaptive behavior. Collectively, these observations show that emotion and cognition are deeply interwoven in the fabric of the brain, suggesting that widely held beliefs about the key constituents of ‘the emotional brain’ and ‘the cognitive brain’ are fundamentally flawed.

Developing a deeper understanding will require a greater emphasis on (a) assessing the real-world relevance of laboratory assays, including measures of brain activity; (b) a network approach to characterizing the neurobiology of emotion–cognition interactions, and (c) mechanistic research. Adopting these strategies mandates collaboration among researchers from different disciplines, with expertise in different species, populations, measurement tools, analytic strategies, and conceptual approaches.

Addressing the interplay of emotion and cognition is a matter of theoretical as well as practical importance. In particular, many of the most common and costly neuropsychiatric disorders—anxiety, depression, schizophrenia, substance abuse, chronic pain, autism, and so on—involve prominent disturbances of cognition *and* emotion (Millan, 2013). Fundamentally, they are disorders of the emotional-cognitive brain. Collectively, these disorders far outstrip the global burden of cancer or cardiovascular disease (Collins et al., 2011; Whiteford et al., 2013; DiLuca and Olesen, 2014), underscoring the importance of accelerating efforts to understand the neural systems underlying the interaction and integration of emotion and cognition.

## GLOSSARY OF TERMS NOT DEFINED IN THE MAIN TEXT

**Affect:** The experience or expression of emotion (see also Barrett et al., 2007).

**Anxiety:** A sustained state of heightened apprehension in response to uncertain, distal, or diffuse threat (Davis et al., 2010).

**Cognition:** Cognition is a fuzzy category that conventionally includes processes involved in knowing or ‘thinking,’ including attention, imagination, language, learning, memory, and perception (for discussion, see Duncan and Barrett, 2007).

**Emotion:** Like ‘cognition,’ ‘emotion’ is a fuzzy, contentious category that conventionally includes valenced processes (e.g., action tendencies, attention, overt behavior, subjective feelings, and alterations in peripheral physiology) that are triggered by specific external or internal stimuli (e.g., actual or remembered threat for fear); often taken to include states of anger, disgust, fear, happiness, and sadness (e.g., Ekman and Davidson, 1994; Duncan and Barrett, 2007; Gendron and Barrett, 2009; LeDoux, 2012, 2014).

**Mood:** A low-intensity emotional state that persists in the absence of an explicit triggering stimulus (Ekman and Davidson, 1994).

**Motivation:** Internal states that are elicited by reinforcers and serve to organize behavioral direction (i.e., approach or avoidance) and intensity. Emotional states involve alterations in motivation (e.g., increased avoidance in the case of fear). However, motivation can be altered by homeostatic processes, such as hunger and satiety, that are not conventionally considered emotional (Rolls, 1999).

**Neuroticism/Negative Emotionality:** A fundamental dimension of childhood temperament and adult personality; individuals with high levels of Neuroticism/Negative Emotionality are susceptible to more intense or long-lasting negative emotions, including anger, anxiety, fear, guilt, and sadness (Watson and Clark, 1984; Caspi et al., 2005).

**Reinforcer:** Rewards and punishments; anything an organism will work to approach or avoid (Rolls, 1999).

## AUTHOR CONTRIBUTIONS

All the authors supervised the Special Research Topic. Hadas Okon-Singer and Alexander J. Shackman wrote the paper. All the authors edited and revised the paper.

## Conflict of Interest Statement

The authors declare that the research was conducted in the absence of any commercial or financial relationships that could be construed as a potential conflict of interest.
